# PGC-1α limits angiotensin II-induced rat vascular smooth muscle cells proliferation via attenuating NOX1-mediated generation of reactive oxygen species

**DOI:** 10.1042/BSR20150112

**Published:** 2015-09-29

**Authors:** Qingbin Zhao, Junfang Zhang, Huifang Wang

**Affiliations:** * Department of Geratology, the First Affiliated Hospital of Xi'an Jiaotong University, Xi'an, Shanxi 710061, China; † Department of Internal Medicine, Chencang Hospital, Baoji, Shanxi 721300, China

**Keywords:** angiotensin II, NADPH, NOX1, PGC-1α, proliferation, reactive oxygen species

## Abstract

AngII (angiotensin II)-induced excessive ROS (reactive oxygen species) generation and proliferation of VSMCs (vascular smooth muscle cells) is a critical contributor to the pathogenesis of atherosclerosis. PGC-1α [PPARγ (peroxisome-proliferator-activated receptor γ) co-activator-1α] is involved in the regulation of ROS generation, VSMC proliferation and energy metabolism. The aim of the present study was to investigate whether PGC-1α mediates AngII-induced ROS generation and VSMC hyperplasia. Our results showed that the protein content of PGC-1α was negatively correlated with an increase in cell proliferation and migration induced by AngII. Overexpression of PGC-1α inhibited AngII-induced proliferation and migration, ROS generation and NADPH oxidase activity in VSMCs. Conversely, Ad-shPGC-1α (adenovirus-mediated PGC-1α-specific shRNA) led to the opposite effects. Furthermore, the stimulatory effect of Ad-shPGC-1α on VSMC proliferation was significantly attenuated by antioxidant and NADPH oxidase inhibitors. Analysis of several key subunits of NADPH oxidase (Rac1, p22*^phox^*, p40*^phox^*, p47*^phox^* and p67*^phox^*) and mitochondrial ROS revealed that these mechanisms were not responsible for the observed effects of PGC-1α. However, we found that overexpression of PGC-1α promoted NOX1 degradation through the proteasome degradation pathway under AngII stimulation and consequently attenuated NOX1 (NADPH oxidase 1) expression. These alterations underlie the inhibitory effect of PGC-1α on NADPH oxidase activity. Our data support a critical role for PGC-1α in the regulation of proliferation and migration of VSMCs, and provide a useful strategy to protect vessels against atherosclerosis.

## Abstract (precis)

The protein content of PGC-1α was negatively correlated with an increase in cell proliferation and migration induced by AngII. PGC-1α could decrease ROS generation derived from NADPH oxidase induced by AngII, thus attenuating VSMC hyperplasia.

## INTRODUCTION

During the progression and development of atherosclerosis, VSMCs (vascular smooth muscle cells) proliferates and migrates to the bulk of extracellular matrix, resulting in the thickening of the artery wall and exacerbating atherosclerotic lesions [1,2]. VSMC hyperplasia caused by abnormal proliferation and migration is a major contributor to the development of atherosclerosis [3]. Increasing evidence has shown that AngII (angiotensin II) plays an important role during proliferation in VSMCs [4,5]. AngII activates diverse signalling responses via binding the AT_1_R (AngII type 1 receptor), consequently mediating ROS (reactive oxygen species), proliferation and migration [6–8]. However, the mechanisms by which AngII induces the excessive proliferation of VSMC have not been fully elucidated.

NADPH oxidase is a principal factor in AngII-induced ROS generation [9], which is an essential contributor to cell proliferation and atherosclerosis [10]. The NADPH oxidase comprises the several main isoforms of ROS-generating enzymes: two plasma membrane subunits NOX2 (NADPH oxidase 2)/gp91*^phox^* and p22*^phox^*, and four cytosolic subunits p40^phox^, p47*^phox^*, p67*^phox^* and Rac1 [11]. These subunits differ not only in their capacity to generate ROS, but also in their distribution in specific cell types. Notably, VSMCs lack NOX2, but express the NOX2 homologue NOX1 [12,13]. However, whether NOX1 is involved in the regulation of VSMC proliferation remains unknown.

PGC-1α [PPARγ (peroxisome-proliferator-activated receptor γ) co-activator-1α] is considered to be the main regulator of the expression of many mitochondrial proteins and energy metabolism [14,15]. PGC-1α regulates cell differentiation [16], gluconeogenesis [17], neuron excitability and ROS generation [18]. Interestingly, it has been documented to be a broad and powerful regulator of ROS metabolism, because reduction in PGC-1α paralleled with a decrease in antioxidant enzymes [18]. Moreover, a study in VSMCs by overexpression and knockdown strategies demonstrated that PGC-1α inhibited high-glucose-induced VSMC proliferation [19].

In the present study, we therefore sought to determine whether PGC-1α plays a vital role in AngII-induced ROS-mediated VSMC proliferation and migration, and to explore further the potential mechanisms.

## MATERIALS AND METHODS

### Materials and reagents

DMEM (Dulbecco's modified Eagle's medium)/Ham's F12, FBS and MitoSOX Red reagent were purchased from Invitrogen. AngII, Crystal Violet, NAC (*N*-acetyl-L-cysteine), antimycin A, ML171 (2-acetylphenothiazine), lucigenin, NADPH and CHX (cycloheximide) were purchased from Sigma Chemical Co. Chloroquine, MG132 and protease and phosphatase inhibitor cocktail were from Calbiochem. Anti-p-p47*^phox^* (Ser^370^) antibody was obtained from Assay Biotechnology. Antibodies targeting p47*^phox^* and GAPDH (glyceraldehyde-3-phosphate dehydrogenase) were purchased from Cell Signaling Technology. Antibodies against PGC-1α, total Rac1 and p22*^phox^* were from Santa Cruz Biotechnology. Antibodies targeting p40*^phox^*, p67*^phox^* and NOX1 were obtained from Abcam.

Male Sprague–Dawley rats 3–4 weeks of age were supplied by the Experimental Animal Center of Xi'an Jiaotong University, Xi'an, Shanxi, China. All animal experiments were approved by the Committee on the Ethics of Animal Experiments of Xi'an Jiaotong University.

### Cell culture

Primary VSMCs were isolated from rat thoracic aortas as described previously [19]. The cells were maintained in DMEM/Ham's F12 supplemented with 20% (v/v) FBS at 37°C in a humidified incubator supplemented with 5% CO_2_. All experiments were conducted using VSMCs between passages 4 and 8 that were growth-arrested at 70% confluence for 48 h with medium containing 0.1% FBS.

### [^3^H]thymidine-incorporation assay

VSMCs were seeded in 24-well plates and growth-arrested by incubating in DMEM/Ham's F12 containing 0.1% FBS for 48 h. The quiescent cells were divided into several groups and applied to different treatments, then 1 μCi/ml [^3^H]thymidine was added for 3 h of incubation at 37°C. The incorporation of [^3^H]thymidine into DNA was examined with Beckman liquid-scintillation counter.

### Cell migration assay

The cell migration assay was performed using Transwell chambers with fibronectin-coated 8-μm-pore-size polycarbonate membrane (BD Bioscience). VSMCs subjected to adenovirus infection in the absence or presence of AngII according to the experiment requirements were suspended in DMEM/Ham's F12 containing 0.5% FBS. Then, 600 μl of serum-free medium was added to the lower compartment and 100 μl of cell suspension (4×10^4^ cells/well) was added to the upper compartment. The cells were incubated at 37°C. After 24 h, non-migrated cells on the upper membrane were removed by a cotton swab. The migrated cells were fixed with methanol for 30 min and stained with 0.1% Crystal Violet. Stained migratory cells were photographed under an inverted light microscope and quantified by manual counting (Olympus Corporation) in five randomly selected areas of view. Six independent experiments were performed.

### Pharmacological approaches

NAC (5 mmol/l), ML171 (5 μmol/l), antimycin A (10 μmol/l), rotenone (20 μmol/l), chloroquine (10 μg/ml) or MG132 (10 μg/ml) was added to the cell culture for 1 h before various treatments. For experiments using ML171, chloroquine or MG132, the compound was continued during the stimulation, whereas for other treatments, medium containing the compound was replaced by fresh medium according to the experimental requirements. AngII (0.1 μmol/l) or CHX (10 μg/ml) was added to cells for the indicated time.

### Western blot analysis

VSMCs were lysed in RIPA lysis buffer (Beyotime) containing protease and phosphatase inhibitor cocktail. Protein concentration was determined by Bradford assay (Bio-Rad Laboratories). Equal amounts of protein were separated by SDS/PAGE (6–10% gel) and transferred on to nitrocellulose membranes (Millipore). The membranes were blocked in 5% (w/v) non-fat dried skimmed milk powder in TBS, followed by incubation of the appropriate primary antibodies. After washing with TBS, membranes were incubated with secondary antibodies including HRP (horseradish peroxidase)-conjugated anti-rabbit, anti-mouse or anti-goat antibodies (Cell Signaling Technology), and the bands were visualized using an ECL kit (Beyotime).

### Adenovirus infection of rat VSMCs

Ad-shPGC-1α (adenovirus-mediated PGC-1α-specific shRNA), Ad-PGC-1α (adenovirus-mediated PGC-1α-specific siRNA) and Ad-NOX1 (adenovirus-mediated NOX1-specific siRNA) were purchased from Sunbio Medical Biotechnology. Adenovirus infection of the quiescent VSMCs was performed at different MOIs (multiplicities of infection) in DMEM/Ham's F12 containing 2% (v/v) FBS for 48 h and treated with AngII according to the experiment requirements. LacZ (Clotech) was used as a negative control.

### ROS detection

ROS in VSMCs were visualized by H_2_DCF-DA (2′,7′-dichlorofluorescin diacetate) (Invitrogen) as described previously [18]. VSMCs were seeded in six-well plates or 96-well black-bottomed plates, and then different treatments were applied. After treatment, cells were incubated with H_2_DCF-DA (10 μmol/l) in serum-free DMEM/Ham's F12 for 30 min at 37°C. Then cells were washed twice with PBS and images were taken with a confocal laser-scanning microscope (FV1000, Olympus) at 488 nm excitation and 525 nm emission wavelengths. To quantify the fluorescence intensity of H_2_DCF-DA, stained cells in 96-well plates were quantified using an Infinite F500 Microplate Reader at the same excitation and emission wavelengths and normalized to protein concentrations.

### Detection of mROS (mitochondrial ROS) generation

MitoSOX Red reagent, which is a highly selective mitochondrial dye, was used to measure mROS. In brief, cells were incubated with MitoSOX Red (5 μmol/l) in serum-free DMEM/Ham's F12 in the dark at 37°C for 30 min. Finally, the cells were gently washed with warm PBS and visualized under an Olympus fluorescence microscope.

### Detection of NADPH oxidase activity

NADPH oxidase activity of VSMCs was measured by a lucigenin ECL assay as described previously [20]. Briefly, VSMCs were washed twice with PBS and lysed in NADPH lysis buffer (1.0 mol/l K_2_HPO_4_, 0.1 mol/l EGTA, 0.15 mol/l and protease inhibitor cocktail, pH 7.0) and sonicated with three cycles of 6-s bursts on ice. After centrifugation at 12000 ***g*** for 5 min, the supernatant was harvested and incubated with lucigenin (5 mmol/l) for 10 min at 37°C in the dark. The basal RLU (relative light units) of chemiluminescence were determined using a luminometer (Promega). NADPH (100 μmol/l) was immediately added to the suspension to start the reaction and the chemiluminescence was determined as experimental RLU. The NADPH oxidase activity was expressed as RLU, which were normalized to protein concentration.

### RNA isolation and analysis

Total RNA from VSMCs was isolated using an RNeasy Micro Kit (Qiagen) according to the manufacturer's instructions. Reverse transcription was performed using the ReverTra ACE qPCR RT Kit (Toyobo) and PCR amplification was carried out with the ABI Prism 7000 sequence detection (Applied Biosystems). Sequence-specific primers and SYBR Green PCR master mix were obtained from Invitrogen. The primer sequences used to amplify rat *Nox1* cDNA were 5′-TCACTAACGTGTGGGTCAGC-3′ (sense) and 5′-GCTCTCATGTTGCCAAAGCC-3′ (antisense); primers for *Nox2* were 5′-TTGGCTGGCATCACAGGAAT-3′ (sense) and 5′-TCAAACTGTCCGAAGTCTGTCC-3′ (antisense); primers for *Nox4* were 5′-CCTCTGTCTGCTTGTTTGGC-3′ (sense) and 5′-TCCTAGGCCCAACATCTGGT-3′ (antisense); primers for *Actb* (β-actin) were 5′-AGGGAAATCGTCGTGCGTGAG-3′ (sense) and 5′-CGCTCATTGCCGATAGT-3′ (antisense). Amplification was carried out for 38 cycles of 95°C for 30 s, 60°C for 1 min and 72°C for 30 s after an initial 5 min at 95°C.

### Rac1 activation assay

The activity of Rac1 was determined using Rac1 activation assay kit (Cell Biolabs) according to the manufacturer's instructions. Proteins were incubated with beads conjugated to PAK (p21-activated protein kinase) PBD (p21-binding domain) for 1 h at 4°C. Beads were boiled in SDS buffer to release active Rac1. The activated Rac1 was detected by Western blotting using a specific anti-Rac1 antibody.

### Statistical analysis

All data are given as means±S.E.M. and analysed by SPSS 17.0 statistical software. For the dosing of AngII, a one-way ANOVA was used. A two-way or three-way ANOVA was used for two-factor or three-factor analysis. The regression analysis was determined using the Pearson correlation test. Values of *P*<0.05 were considered to be statistically significant.

## RESULTS

### AngII-induced VSMC proliferation and migration are negatively correlated with PGC-1α expression

Rat VSMCs were treated with various concentrations of AngII (10^−9^–10^−6^ mol/l) for 48 h. We found that AngII increased cell proliferation and migration in a dose-dependent manner. AngII at 0.1 μmol/l resulted in a nearly 3.4-fold increase in proliferation and a 1.9-fold increase in migration respectively compared with the control group (Figures 1A and 1B).

**Figure 1:**
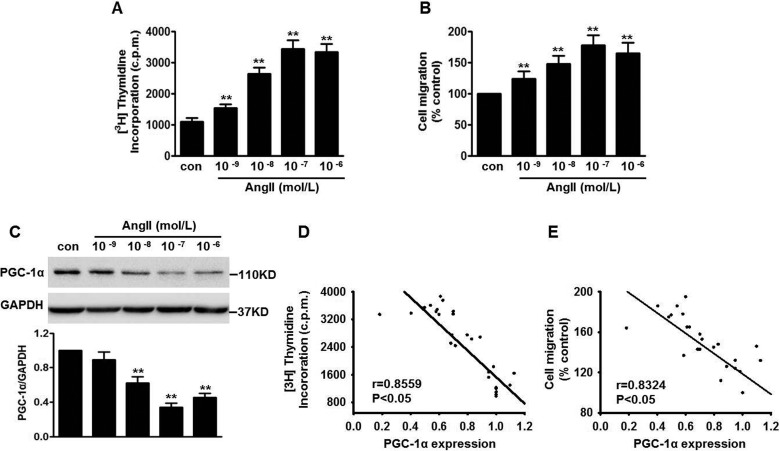
AngII-induced VSMC proliferation and migration is associated with decreased PGC-1α expression (**A**) The quiescent rat VSMCs were incubated with different concentrations of AngII (10^−9^–10^−6^ mol/l) for 48 h. Cells were incubated with [^3^H]thymidine for the last 3 h. Incorporation of [^3^H]thymidine was then determined. (**B**) Quantification of cell migration after AngII challenge. (**C**) Western blot analysis of PGC-1α protein expression. Molecular masses are indicated in kDa. (**D** and **E**) The incorporation of [^3^H]thymidine (**D**) and cell migration (**E**) were negatively correlated with the expression of PGC-1α respectively. **P<0.01 compared with control (*n*=6).

To clarify the relationship between AngII-induced VSMC proliferation and PGC-1α expression, we investigated the effects of different concentrations of AngII on PGC-1α expression in rat VSMCs. As shown in Figure 1(C), incubation of AngII for 48 h decreased PGC-1α protein expression in a dose-dependent manner. PGC-1α expression reached the minimal expression level at 0.1 μmol/l. Therefore 0.1 μmol/l AngII was used in the subsequent experiments. Moreover, the regression analysis showed that PGC-1α expression was negatively correlated with the cell proliferation and migration respectively (Figures 1D and 1E), suggesting that PGC-1α expression may be involved in AngII-induced VSMC proliferation and migration.

### PGC-1α inhibits AngII-induced VSMC proliferation and migration

To investigate further the role of PGC-1 in VSMC proliferation and migration, cells were infected with Ad-shPGC-1α for 48 h. The silencing efficiency was evaluated by Western blotting. PGC-1α protein level showed a 69% decrease after Ad-shPGC-1α infection at 40 MOI compared with the control group. Conversely, PGC-1α protein expression was significantly elevated following overexpression of PGC-1α using Ad-PGC-1α. The maximal expression level of PGC-1α was observed at 20 MOI, whereas LacZ showed no effect (Figure 2A). Therefore we selected Ad-shPGC-1α at 40 MOI and Ad-PGC-1α at 20 MOI for 48 h in the subsequent experiments. [^3^H]thymidine incorporation in VSMCs infected with Ad-PGC-1α or Ad-shPGC-1α was comparable with that in untreated cells (results not shown). Strikingly, the ability of AngII to induce cell proliferation was almost lost when VSMCs were infected with Ad-PGC-1α. However, PGC-1α knockdown dramatically enhanced AngII-induced VSMC proliferation (Figure 2B). Similarly, Transwell analysis showed that the increased number of migrated cells induced by AngII was decreased after PGC-1α overexpression, whereas conditions with PGC-1α knockdown demonstrated the opposite (Figures 2C and 2D). Of note, the enhanced effects of PGC-1α inhibition on AngII-induced cell proliferation and migration were all markedly attenuated by the antioxidant NAC (5 μmol/l).

**Figure 2:**
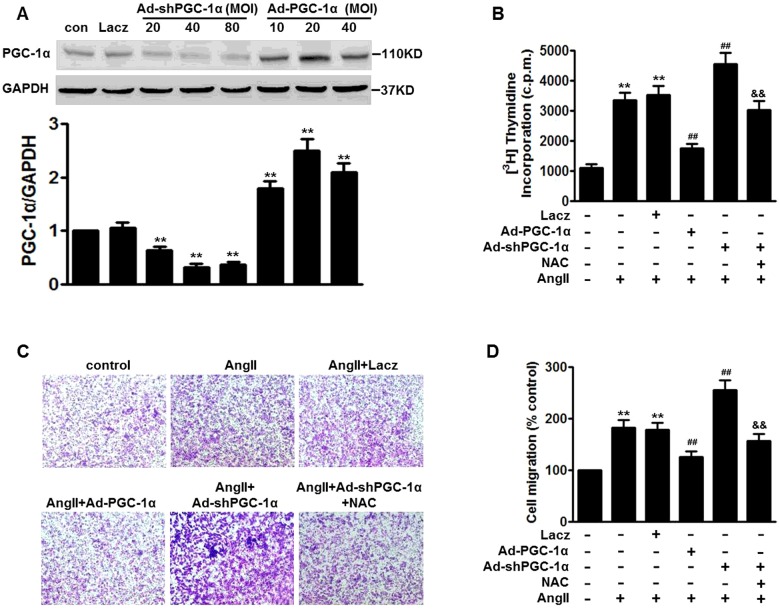
Effects of PGC-1α on AngII-induced VSMC proliferation and migration (**A**) VSMCs were transfected with LacZ (80 MOI), Ad-shPGC-1α (20, 40 and 80 MOI) or Ad-PGC-1α (10, 20 and 40 MOI) for 48 h. Efficiency of infection was confirmed by Western blotting with anti-PGC-1α antibody. Molecular masses are indicated in kDa. (**B**) Cells were pre-treated with NAC (5 mmol/l) for 1 h, followed by transfection of LacZ (40 MOI), Ad-shPGC-1α (40 MOI) or Ad-PGC-1α (20 MOI) for 48 h, and then were submitted to AngII (0.1 μmol/l) conditions for another 48 h. [^3^H]thymidine was added and the incorporation of [^3^H]thymidine was determined at 3 h. (**C**) VSMC migration was examined by Transwell analysis after the treatment in (**B**). Representative images for cell migration assay captured by microscope from six independent experiments (×200 magnification). (**D**) Quantitative analysis of cell migration. **P<0.01 compared with control; ##*P*<0.01 compared with AngII; &&*P*<0.01 compared with Ad-shPGC-1α+AngII (*n*=6).

### PGC-1α limits AngII-induced ROS generation via inhibition of NOX1

We first evaluated the effect of PGC-1α on total ROS generation with the ROS-reactive dye H_2_DCF-DA. Overexpression or inhibition of PGC-1α produced no effect on ROS generation in the absence of AngII (results not shown). AngII treatment significantly increased ROS generation, which was almost abolished by PGC-1α overexpression. In contrast, PGC-1α knockdown further enhanced AngII-induced ROS generation (Figures 3A and 3B). These data indicate the essential role of PGC-1α in the regulation of AngII-induced ROS generation in VSMCs.

**Figure 3:**
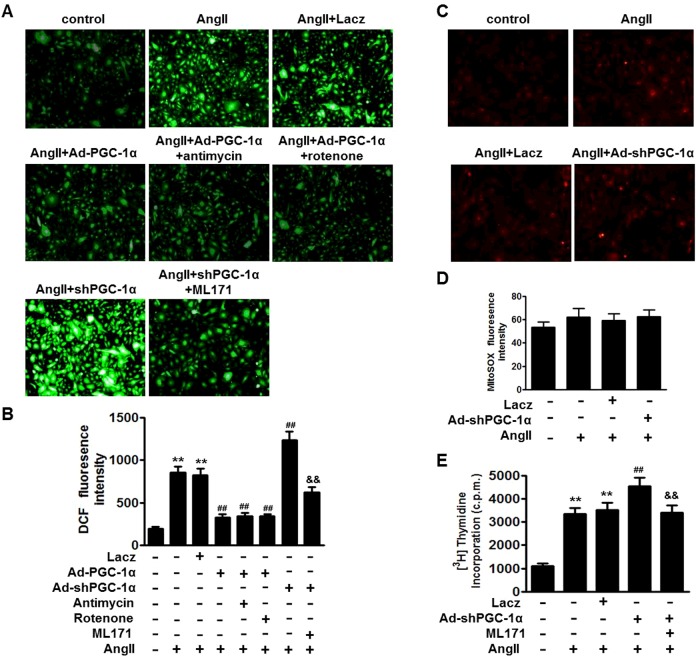
Effects of PGC-1α on AngII-induced ROS generation in VSMCs (**A**) Cells were pre-treated with test compounds as described in the Materials and methods section. Then VSMCs were infected with LacZ, Ad-shPGC-1α or Ad-PGC-1α for 48 h before incubation with AngII (0.1 μmol/l) for another 48 h. Representative images (×200 magnification) for cells loaded with H_2_DCF-DA (10 μmol/l) for 30 min. (**B**) Quantitative analysis of DCF (2′,7′-dichlorofluorescein) fluorescence intensity. (**C**) Confocal microscopy was used to localize mROS generation (×200 magnification). (**D**) Quantitative analysis of MitoSOX fluorescence intensity. (**E**) VSMCs were pre-treated with ML171 (5 μmol/l) for 1 h, and were then infected with LacZ or Ad-shPGC-1α before exposure to AngII (0.1 μmol/l) for 48 h. [^3^H]thymidine was added and the incorporation of [^3^H]thymidine was determined after 3 h. **P<0.01 compared with control; ##*P*<0.01 compared with AngII; &&*P*<0.01 compared with Ad-shPGC-1α+AngII (*n*=6).

Next, we examined how PGC-1α regulates AngII-induced ROS generation in VSMCs. Incubation of VSMCs with mROS-specific stimulators, such as antimycin A and rotenone, did not ablate the inhibitory effect of PGC-1α on ROS generation induced by AngII (Figures 3A and 3B). Although MitoSOX Red fluorescence was higher following AngII exposure, no significant differences were noted across treatment conditions (Figures 3C and 3D). These data exclude the possibility that PGC-1α knockdown accelerates AngII-induced ROS generation via increasing mROS generation. Interestingly, pharmacological inhibition of NOX1 with its selective inhibitor ML171 largely abrogated the elevation of ROS generation in Ad-shPGC-1α-infected AngII-treated cells (Figures 3A and 3B). In addition to NAC, we found that ML171 also remarkably prevented the excessive proliferation in Ad-shPGC-1α-infected AngII-treated cells (Figure 3E). Collectively, these data suggest that NOX1 activation may underlie, at least in part, the enhanced effect of PGC-1α knockdown on ROS generation and cell proliferation.

### PGC-1α attenuates AngII-stimulated NADPH oxidase activity through specifically inhibiting NOX1 expression

Because NOX1 is an important isoform of NADPH oxidase, we investigated whether PGC-1α influences NADPH oxidase activity directly. As shown in Figure 4(A), overexpression of PGC-1α remarkably attenuated the AngII-induced increase in NADPH oxidase activity, whereas blockade of PGC-1α produced the opposite effects. Notably, inhibition of NOX1 with ML171 almost abolished the enhanced effects of PGC-1α knockdown on NADPH oxidase activity. To confirm whether the effects of PGC-1α on NADPH oxidase activity are associated with NOX1, we co-infected VSMCs with Ad-PGC-1α and Ad-NOX1 in the presence of AngII. The successful overexpression of NOX1 expression was confirmed by Western blot analysis and quantitative PCR (Supplementary Figures S1A and S1B). Moreover, overexpression of NOX1 in rat VSMCs did not alter the expression of NOX2 and NOX4 (Supplementary Figure S1B). Following overexpression of NOX1, the inhibitory effect of PGC-1α on NADPH oxidase activity under AngII stimulation was abolished, indicating that NOX1 is a critical molecular target for PGC-1α in the regulation of NADPH oxidase activity.

**Figure 4:**
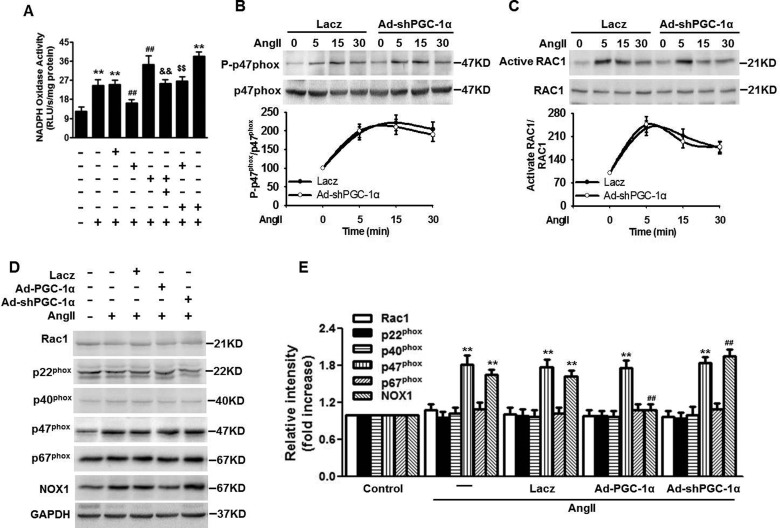
Overexpression of PGC-1α inhibits AngII-induced NOX1 expression (**A**) Quantitation analysis of NADPH oxidase activity measured in the presence of lucigenin (5 mmol/l) followed by treating with or without NADPH (100 μmol/l). (**B** and **C**) VSMCs were treated with LacZ or Ad-PGC-1α for 48 h, and then AngII at 0.1 μmol/l was added for the indicated times. Phospho-p47*^phox^* and total p47*^phox^* (**B**), and active Rac1 and total Rac1 (**C**) were detected by Western blotting. Molecular masses are indicated in kDa. (**D**) Western blot analysis of Rac1, p22*^phox^*, p40*^phox^*, p67*^phox^* and NOX1 in the lysates of VSMCs. Cells were infected with LacZ, Ad-shPGC-1α or Ad-PGC-1α for 48 h before incubation of AngII for another 48 h. GAPDH was used as loading control. Molecular masses are indicated in kDa. (**E**) Densitometric analysis of Rac1, p22*^phox^*, p40*^phox^*, p67*^phox^* and NOX1 was performed. **P<0.01 compared with control; ##*P*<0.01 compared with AngII; &&*P*<0.01 compared with Ad-shPGC-1α+AngII; $$*P*<0.01 compared with Ad-PGC-1α+AngII (*n*=6).

The NADPH oxidase complex in VSMCs is composed of two membrane subunits, NOX2/gp91*^phox^* and p22*^phox^*, and four cytosolic subunits, p40*^phox^*, p47*^phox^*, p67*^phox^* and Rac1 [11,12]. To determine whether PGC-1α regulates NOX1 specifically, the activation or expression of these subunits was measured. In PGC-1α-knockdown VSMCs, the phosphorylation of p47*^phox^* and activation of Rac1 were comparable with those of LacZ-infected cells under AngII stimulation (Figures 4B and 4C). The expression of Rac1, p22*^phox^*, p40*^phox^* and p67*^phox^* remained unchanged across the different treatment conditions. Although p47*^phox^* protein expression was elevated following exposure to AngII, few significant changes were observed after overexpression or knockdown of PGC-1α. Of note, NOX1 expression was strikingly increased after AngII treatment. Moreover, following PGC-1α overexpression, NOX1 expression was no longer elevated by AngII. In contrast, the increased NOX1 expression was augmented further in Ad-shPGC-1α-infected cells (Figures 4D and 4E).

### PGC-1α decreases NOX1 expression induced by AngII through the proteasome degradation pathway

To investigate further the mechanism by which PGC-1α inhibits AngII-induced NOX1 expression, we first determined the expression level of *NOX1* mRNA. Although the *NOX1* mRNA level was increased following AngII treatment, we did not detect any differences after overexpression or knockdown of PGC-1α (Figure 5A). These results suggest that post-transcriptional regulation seems to be taking place. We next examined the stability of NOX1 protein by using a protein synthesis inhibitor, CHX (10 μg/ml). Under AngII stimulation, CHX treatment showed a time-dependent decrease in NOX1 protein level in VSMCs. Compared with the LacZ group, overexpression of PGC-1α significantly increased the rate of NOX1 degradation, shortening NOX1 protein half-life by more than 3 h (Figure 5B). These results indicate that PGC-1α participates in the degradation of NOX1 protein. Previous studies have suggested that the proteasome-dependent endoplasmic reticulum-associated degradation pathway and lysosome-associated degradation pathway are involved in NOX1 protein degradation [11,21,22]. Therefore cells were pre-treated with the lysosome blocker chloroquine or the proteasome inhibitor MG132 followed by Ad-PGC-1α infection in the absence or presence of AngII, and then the NOX1 protein level was analysed by Western blotting. Neither of these pharmacological approaches had an effect on NOX1 expression in the absence of AngII. Notably, the inhibitory effect of Ad-PGC-1α on NOX1 expression in the presence of AngII was dramatically restored by MG132, but not chloroquine (Figures 5C and 5D). Collectively, these data suggest that PGC-1α inhibits AngII-stimulated NOX1 expression by enhancing NOX1 degradation through the proteasome degradation pathway.

**Figure 5:**
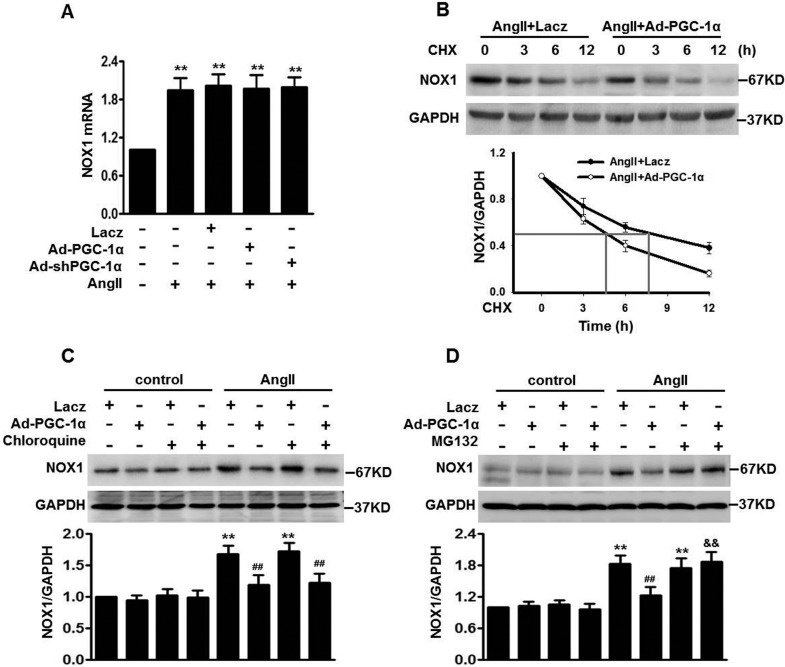
PGC-1α accelerates proteasome-mediated degradation of NOX1 protein under AngII conditions (**A**) *NOX1* mRNA expression in VSMCs infected with LacZ, Ad-shPGC-1α or Ad-PGC-1α for 48 h before incubation of AngII for another 48 h. **P<0.01 compared with control group (*n*=6). (**B**) Cells were infected with LacZ or Ad-PGC-1α in the presence of AngII, and then CHX (10 μg/ml) was added for the indicated time. NOX1 expression was analysed by Western blotting. Molecular masses are indicated in kDa. A 50% reduction of NOX1 protein level is represented by grey lines. (**C** and **D**) Western blot analysis of NOX1 expression in VSMCs pre-treated with chloroquine (10 μg/ml) (**C**) or MG132 (10 μg/ml) (**D**) for 1 h and then infected with LacZ or Ad-PGC-1α in the absence or presence of AngII. Molecular masses are indicated in kDa. **P<0.01 compared with LacZ alone; ##*P*<0.01 compared with LacZ+AngII; &&*P*<0.01 compared with Ad-PGC-1α+AngII (*n*=6).

## DISCUSSION

Atherosclerosis is the primary cause of heart disease and stroke, which is characterized by smooth muscle proliferation and thickening of the arterial wall [10,23]. Accumulating evidence suggests that AngII is the most potent stimulator of VSMC proliferation [4,7]. Therefore an understanding of the precise molecular mechanism of AngII-induced VSMC proliferation is essential to elucidate the pathological process of atherosclerosis. PGC-1α is an important regulator in mitochondrial function and energy metabolism. A previous study in HeLa cells showed that inhibition of PGC-1α may contribute to mitochondrial dysfunction [24]. Overexpression of PGC-1α attenuated ischaemia/reperfusion-induced cardiac contractility in heart [25], whereas a lack of PGC-1α in mice contributed to the development of cardiac dysfunction [15]. On the other hand, a decrease in PGC-1α expression was observed in adipose tissue from insulin-resistant subjects [26], as well as in skeletal muscle from Type 2 diabetics [27]. Similarly, both mRNA and protein levels of PGC-1α were significantly reduced after high glucose challenge [19]. These results suggest that the alterations in PGC-1α expression are causally associated with the corresponding physiological and pathological processes. In the present study, we were the first, to our knowledge, to demonstrate that AngII decreases PGC-1α expression in rat VSMCs. We found that PGC-1α protein expression was inhibited by AngII dose-dependently and negatively correlated with cell proliferation and migration. Furthermore, overexpression of PGC-1α abolished, whereas knockdown of PGC-1α enhanced, AngII-induced VSMC proliferation and migration. These data indicate that PGC-1α may function as a key regulator in VSMC proliferation.

Next, we explored the mechanism by which PGC-1α regulates VSMC proliferation and migration. Although a number of factors contribute to VSMC hyperplasia, it is likely that oxidative stress is a major underlying mechanism [28]. In the present study, we found that the stimulatory effect of Ad-shPGC-1α on proliferation and migration was abolished by the antioxidant NAC, demonstrating that oxidative stress is involved in PGC-1α-mediated VSMC growth and movement. Of note, in addition to energy metabolism, PGC-1α can induce antioxidant defences and ROS metabolism [18,29]. For example, induction of PGC-1α increased MnSOD (manganese superoxide dismutase) protein and catalase activity in skeletal muscle, which in turn reduced muscle protein oxidation [30]. Consistent with these studies, we found that the excessive ROS generation induced by AngII was attenuated after overexpression of PGC-1α. ROS have been suggested to be involved a number of pathological processes, including hypertension and atherosclerosis [28]. Mitochondrial electron transport chain and NADPH oxidase are the main sources of intracellular ROS [31,32]. Interestingly, we found that mROS-specific stimulators did not change the inhibitory effect of PGC-1α overexpression on ROS generation. These results, together with the mROS generation (MitoSOX staining), demonstrated that mROS generation is not responsible for the increased ROS generation after PGC-1α inhibition. However, NOX1 inhibitor abolished the stimulatory effect of Ad-shPGC-1α on ROS generation, indicating that NADPH oxidase is the main source of ROS generation during this process. This was confirmed by NADPH oxidase activity analysis.

To explain how PGC-1α regulates NADPH oxidase activity, we examined the protein content of the main subunits of NADPH oxidase. In the present study, we showed that the effect of PGC-1α on NADPH oxidase activity was not associated with the protein expression of RAC1, p22*^phox^*, p40*^phox^*, p47*^phox^* or p67*^phox^*, as well as the phosphorylation of p47*^phox^* or activation of Rac1, suggesting that these subunits may be not the molecular targets for PGC-1α in the regulation of NADPH oxidase activity. Nevertheless, NOX1 shares 56% similarity with NOX2 and is abundantly expressed in specific cell types, such as VSMCs [13]. In the present study, we found that shRNA-mediated knockdown of PGC-1α enhanced AngII-induced NOX1 expression, whereas overexpression of PGC-1α alleviated it. Moreover, the inhibitory effect of PGC-1α on NADPH oxidase activity was abolished following overexpression of NOX1. These results indicate that changes to NOX1 expression probably specifically account for the effect of PGC-1α on ROS generation and proliferation.

We found that neither up-regulation nor down-regulation of PGC-1α changed the mRNA level of PGC-1α, indicating that transcriptional regulation is not involved. CHX treatment showed that overexpression of PGC-1α accelerated NOX1 degradation in the presence of AngII, suggesting that degradation of NOX1 protein seems to be taking place. Accumulating evidence has found that isoforms of the NOX family could be degraded quickly via a proteasome-dependent endoplasmic reticulum-associated degradation pathway. However, other mechanisms, such as the lysosome-associated degradation pathway, may occur when NOXs translocate from the endoplasmic reticulum to plasma membranes [11,21,22]. In the present study, we found that treatment with proteasome inhibitor restored Ad-PGC-1α-decreased NOX1 expression, whereas a lysosome blocker produced no reversible effect. Together, our data suggest that PGC-1α promotes NOX1 degradation via the proteasome degradation pathway under AngII stimulation, and consequently attenuates NOX1 expression.

In summary, we have demonstrated the correlation between PGC-1α expression and VSMC proliferation. Our results reveal that PGC-1α could decrease ROS generation derived from NADPH oxidase, thus attenuating AngII-induced VSMC hyperplasia. These findings suggest that elevation of PGC-1α may be a novel strategy to prevent the development of atherosclerosis, and support further investigation of PGC-1α expression in vascular diseases.

## Abbreviations

Ad-NOX1: adenovirus-mediated NOX1-specific siRNA
Ad-PGC-1α: adenovirus-mediated PGC-1α-specific siRNA
Ad-shPGC-1α: adenovirus-mediated PGC-1α-specific shRNA
AngII: angiotensin II
CHX: cycloheximide
DMEM: Dulbecco's modified Eagle's medium
GAPDH: glyceraldehyde-3-phosphate dehydrogenase
H_2_DCF-DA: 2′,7′-dichlorofluorescin diacetate
MOI: multiplicity of infection
mROS: mitochondrial ROS
NAC: *N*-acetyl-L-cysteine
NOX: NADPH oxidase
PGC-1α: PPARγ (peroxisome-proliferator-activated receptor γ) co-activator-1α
RLU: relative light unit(s)
ROS: reactive oxygen species
VSMC: vascular smooth muscle cell
